# Radiomic analysis of the proximal femur in osteoporosis women using 3T MRI

**DOI:** 10.3389/fradi.2023.1293865

**Published:** 2023-11-21

**Authors:** Dimitri Martel, Anmol Monga, Gregory Chang

**Affiliations:** Bernard and Irene Schwartz Center for Biomedical Imaging, Department of Radiology, New York University Grossman School of Medicine, New York, NY, United States

**Keywords:** osteoporotic fragility fractures, radiomic analysis, high resolution magnetic resonance imaging, fracture risk, textural feature, bone health assessment

## Abstract

**Introduction:**

Osteoporosis (OP) results in weak bone and can ultimately lead to fracture. MRI assessment of bone structure and microarchitecture has been proposed as method to assess bone quality and fracture risk *in vivo*. Radiomics provides a framework to analyze the textural information of MR images. The purpose of this study was to analyze the radiomic features and its abilityto differentiate between subjects with and without prior fragility fracture.

**Methods:**

MRI acquisition was performed on *n *= 45 female OP subjects: 15 with fracture history (Fx) and 30 without fracture history (nFx) using a high-resolution 3D Fast Low Angle Shot (FLASH) sequence at 3T. Second and first order radiomic features were calculated in the trabecular region of the proximal femur on T1-weighted MRI signal of a matched dataset. Significance of the feature’s predictive ability was measured using Wilcoxon test and Area Under the ROC (AUROC) curve analysis. The features were correlated DXA and FRAX score.

**Result:**

A set of three independent radiomic features (Dependence Non-Uniformity (DNU), Low Gray Level Emphasis (LGLE) and Kurtosis) showed significant ability to predict fragility fracture (AUROC DNU = 0.751, *p* < 0.05; AUROC LGLE = 0.729, *p* < 0.05; AUROC Kurtosis = 0.718, *p* < 0.05) with low to moderate correlation with FRAX and DXA.

**Conclusion:**

Radiomic features can measure bone health in MRI of proximal femur and has the potential to predict fracture.

## Introduction

Osteoporosis (OP) is a disease of increased fracture risk (Fx) caused by reduced bone mass and microarchitectural deterioration of bone tissue. The main consequence of OP is fragility fracture. In 2014, 432,000 hospital admissions, 2.5 million hospital visits and 180,000 nursing home admissions in the USA were attributed to osteoporosis in USA (1). Hip fracture accounts for 72% of all osteoporosis-based fracture costs (1) and results in a mortality as high as 21% in the first year after fracture (2). Given the aging U.S and world population and the prevalence osteoporosis in older individuals, the cost of osteoporosis and fracture care is only expected to increase, further burdening societal healthcare systems (3).

To mitigate the effects of osteoporosis-related fracture, a major step is to improve accurate quantification of fragility fracture risk to make a relevant clinical or pharmaceutical intervention (4). The most common standard-of-care methods to quantify fracture risk are areal bone mineral density (BMD) and trabecular bone score (TBS) calculated using dual energy x-ray absorptiometry (DXA) and the Fracture Risk Assessment Tool (FRAX), a clinical-outcome-based 10-year fracture risk predictor. BMD is the WHO reference standard method used to diagnose osteoporosis (5). However, more than 50% of all fragility fracture cases occur in subjects who do not meet BMD criterion for osteoporosis (*T*-score < −2.5). This indicates that BMD has low sensitivity to diagnose osteoporosis and does not completely capture fracture risk. TBS was developed to estimate bone microarchitectural information. However, it is limited to the lumbar spine and is computed from a 2D projection of trabecular bone microarchitecture (TBA) and does not capture 3D information. FRAX on the other hand considers clinical factors (age, gender, BMI, parental Fx History 6) in its prediction of fracture risk. It is combined with other bone health measures like BMD, which capture bone density but no microarchitectural properties of the bone (6, 7).

Magnetic resonance imaging (MRI) allows quantitative assessment of TBA and was first described two decades ago in the distal radius and calcaneus and more recently has been described in the proximal femur (8–12). MRI of TBA consists of depicting the unique 3-D network, size, and shape of individual submillimeter trabeculae. This requires resolution to be on the order of the size of trabeculae. The proximal femur is relatively deep in the human body, which makes high-resolution imaging more challenging because SNR decays quickly as the distance from the receive coil increases. Only recently, through pulse sequence and coil optimization, the femur was made accessible for trabecular bone analysis.

In addition, MRI of TBA is not widely available since it requires specialized analysis software and intense computation.

Radiomics has been used extensively in cancer research (13–19) and pancreatitis detection (19) and provide a way to construct models based on image analysis. Radiomics image analysis software are widely available and may provide another means to quantify bone microarchitecture on MR images, specifically by analyzing texture, shape, and intensity distribution in the region of interest (20, 21).

The purpose of our study was to use radiomic to measure textural features in the trabecular bone architecture of the proximal femur and determine their relationship with fracture status and compared it to FRAX.

## Material/methods

### Subjects

This prospective, HIPAA compliant study was approved by our institutional review board, and written informed consent was obtained from all subjects. Forty-five postmenopausal women were recruited from our institution with total hip dual-energy x-ray absorptiometry (DXA, GE Lunar, Rahmay, NJ) results consistent with osteoporosis (femoral neck or total hip BMD T scores of greater than −2.5, 15 of whom had radiographically confirmed fragility fractures and 30 of whom did not have a fracture). Fragility fracture was defined as a low-energy fracture due to trauma from a fall of standing height or less. There are subjects that have had more than one fragility fractures, the types of fragility fractures included major osteoporotic fractures of the wrist (*n* = 3), spine (*n* = 2), elbow (*n* = 3), rib (*n* = 3), metatarsal (*n* = 2) and distal radius (*n* = 2). The median time since fragility fracture was 13 months. All subjects were able to ambulate without limitation. FRAX score was computed according to the standard method (https://www.sheffield.ac.uk/FRAX/tool), considering patient race and with/without BMD (total hip BMD *T*-score = −2.26 ± 0.65; Femoral Neck BMD *T*-score = −2.52 ± 0.64). Subjects were divided into two age-matched (age > 40 years) groups: with history of fragility fractures (Fx, *n* = 15) and without (nFx, *n* = 30).

### Magnetic resonance imaging

The non-dominant proximal femur of each subject was scanned on a 3 T MRI scanner (SKYRA system, Siemens Healthcare) using an 26 element receive-coil setup (18 elements from a body matrix coil anteriorly and eight elements from a spine coil posteriorly). The coil was wrapped around the hip and secured by sandbags laterally and a velcro strap. We used a 3-dimensional (3D) fast low-angle shot sequence (FLASH) with the following scan parameters: repetition time (TR)/echo time (TE) = 37 ms/4.92 ms, 0.234 mm × 0.234 mm, slice thickness = 1.5 mm, 60 coronal slices, bandwidth = 130 Hz/pixel, parallel acceleration [generalized autocalibrating partially parallel acquisition (GRAPPA) factor = 2, and acquisition time = 15 min 18 s. The imaging parameters were chosen in order to have the smallest voxel size possible while maintaining high enough SNR to visualize trabeculae and, most importantly, perform the image analysis (minimum of SNR ∼10–15 required).

### Segmentation

**Figure 1:**
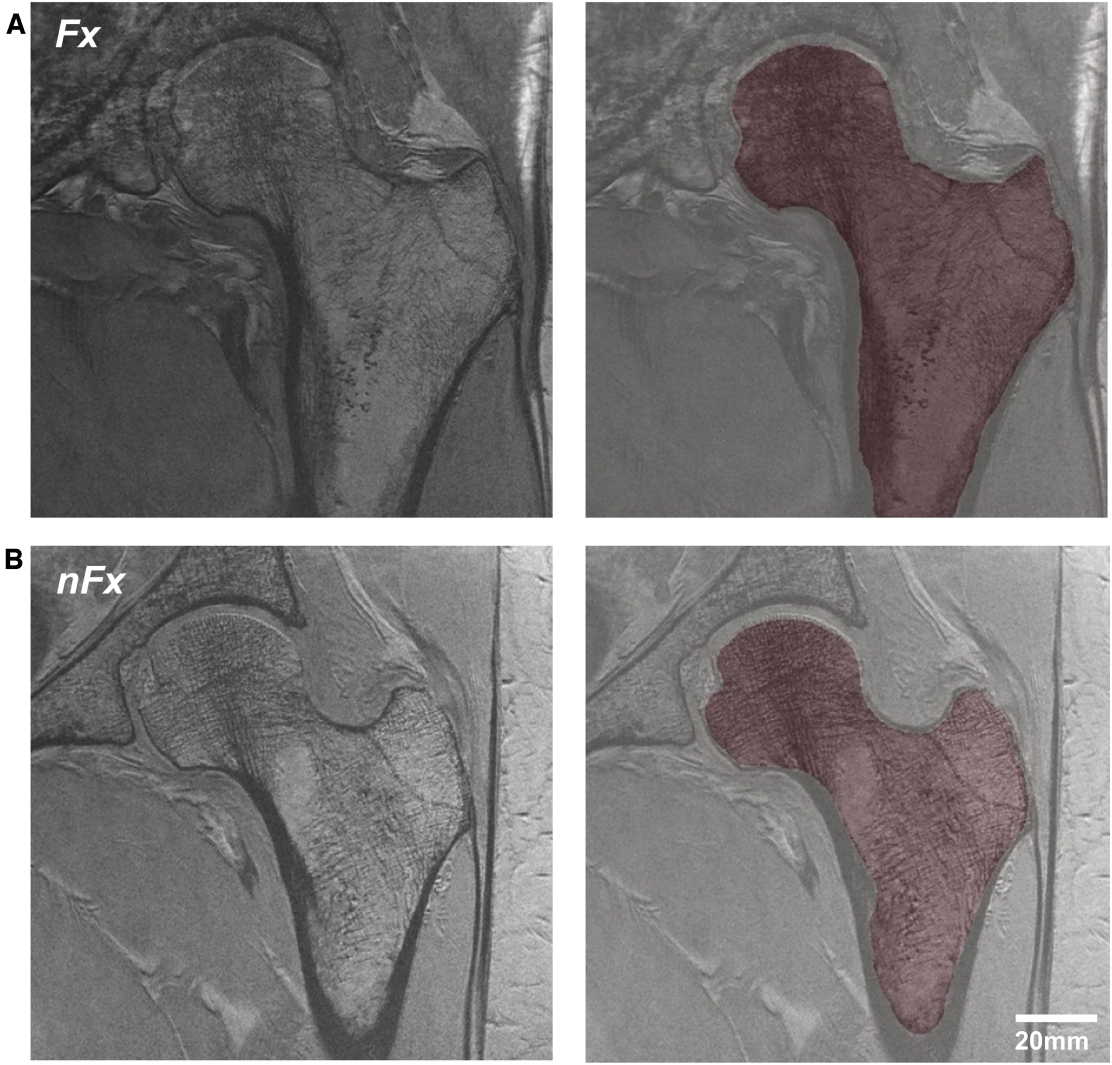
MRI acquisition of the proximal femur and segmented trabecular region in osteoporosis patients, differentiated by (**A**) history of previous fracture and (**B**) no fracture history.

Figure 1 illustrates a typical acquisition of the proximal femur and the segmented trabecular region used for analysis. The segmentation of the proximal femur was conducted by an expert, who manually delineated the trabecular border of bone on MR images using the FireVoxel software package (NYU Center for Advanced Imaging Innovation and Research, New York, USA; https://wp.nyu.edu/firevoxel/downloads/). This expert operated under the direct supervision and guidance of a musculoskeletal radiologist. Subsequently, the region of interest was resampled to an isotropic resolution of 1 × 1 × 1 mm^3^ using 3rd order B-spline interpolation

### Processing

Radiomic textural features of the trabecular region of the proximal femur were extracted using the PyRadiomics toolbox (22). The features encompassed: (1) first-order textural features such as average, contrast, variance, median, skewness, etc., and (2) second-order features like Gray Level Co-occurrence Matrix (GLCM) features, Gray Level Run length Matrix (GLRLM) features, Gray Level Dependence Matrix (GLDM) features, Gray Level Size Zone Matrix (GLSZM) features, and Neighboring Gray Tone Difference Matrix (NGTDM). Second-order features gauge relationships between adjacent pixels. For computation, parameters such as the neighborhood radius (d) and the intensity quantization interval (BW) were considered. The radiomic features were computed for *d* ranging from 1 to 5 and *BW* ranging from 2 to 16.

### Statistical analysis

The association between radiomic features and bone health was discerned using three analysis methods:

1. Wilcoxon test: Assesses feature separability between the Fx and nFx groups, considering features with *p*-value < 0.05 as significantly separable.
2. ROC analysis: Evaluates the capability of radiomic features in predicting fragility fractures, measuring the Area Under the ROC (AUROC). The significance of AUROC values was ascertained through the Wilcoxon test.
3. Spearman correlation: Determines correlations between radiomic measurements and established clinical and imaging parameters, such as age, BMI, FRAX scores (both overall and specific to the hip, and with or without BMD consideration), and BMD values from DXA (T-scores for the hip and femoral neck regions). A *p*-value < 0.05 was considered indicative of significance.

Furthermore, inter-feature correlations among radiomic features were assessed to eliminate redundant features, utilizing Pearson correlation with a significance threshold of *p*-value < 0.001.

## Result

### Subject demographics

Demographic data are presented in Table 1. Age, weight, height, BMI and *T*-scores did not significantly differ between Fx and nFx patients.

**Table 1:** Demographics and characteristics.

	Fx (*n* = 30,F)			nFx (*n* = 15,F)			*p*-value
	Mean	sd	95% CI	Mean	sd	95% CI	
Age	63.13	7.62	58.91–67.36	63.57	6.08	61.29–65.84	0.79
T-score (Femoral Neck)	−2.63	0.85	(−3.10)–(−2.16)	−2.47	0.53	(−2.67)–(−2.28)	0.87
T-score (Hip)	−2.39	0.85	(−2.91)–(−1.88)	−2.19	0.52	(−2.40)–(−1.97)	0.35
Height (m)	1.59	0.08	1.54–1.63	1.57	0.06	1.55–1.60	0.93
Weight (Kg)	53.67	6.31	50.18–57.16	51.30	7.15	48.58–54.02	0.26
BMI (Kg/m^2^)	21.19	1.97	20.10–22.29	20.72	2.38	19.84–21.61	0.44

### Feature selection

Features with significant relationships between radiomic textural features and bone health are presented with their associated abbreviations in Table 2. These features are discriminative of Fx and nFx group or are significantly correlated to FRAX and BMD. The parameter d and BW are optimized for each radiomic feature to produce the most predictive measure of bone health.

**Table 2:** Selected features with significant separability between Fx and nFx group; or significant correlation with FRAX or BMD; and associated abbreviation.

Features	Type	Distance	Bin width	Abbreviation
Dependence Non Uniformity	GLDM	2	4	DNU
Size Zone Non Uniformity	GLSZM	1	2	SZNU
Low Gray Level Emphasis	GLDM	1	8	LGLE
Run Length Non Uniformity	GLRLM	1	16	RLNU
Kurtosis	Firstorder			Kurtosis
Large Dependence Low Gray Level Emphasis	GLDM	5	8	LDLGLE
Long Run Low Gray Level Emphasis	GLRLM	1	8	LRLGLE
Maximum Probability	GLCM	2	1	MP
Low Gray Level Run Emphasis	GLRLM	1	4	LGLE
Large Area Low Gray Level Emphasis	GLSZM	1	1	LALGLE
Gray Level Non Uniformity	GLSZM	1	8	GLNU
Energy	Firstorder			Energy
Total Energy	Firstorder			TE
Coarseness	NGTDM	5	2	Coarseness
Short Run Low Gray Level Emphasis	GLRLM	1	2	SRLGLE
Skewness	Firstorder			Skewness
Zone Variance	GLSZM	1	4	ZV
Cluster Shade	GLCM	1	8	CS
MCC	GLCM	1	1	MCC
Cluster Prominence	GLCM	4	1	CP
Small Dependence Low Gray Level Emphasis	GLDM	4	16	SDLGLE
Busyness	NGTDM	3	16	Busyness

### Feature discrimination

Table 3 shows AUROC of radiomics parameters and clinical parameters able to discriminate between subjects with and without fragility fracture determined in Table 2.

**Table 3:** Separability of feature evaluated through Wilcoxon test and area under the receiver operating characteristic curve (AUROC).

	Fx		nFx		*p*-value	AUROC
Features	Median	IQR	Median	IQR		
DNU	1.070 × 10^5^	9.74 × 10^4^–1.37 × 10^5^	9.100 × 10^4^	8.59 × 10^4^−1.12 × 10^5^	0.007	**0**.**751**
SZNU	4.240 × 10^5^	3.85 × 10^5^–4.82 × 10^5^	3.560 × 10^5^	3.36 × 10^5^–4.19 × 10^5^	0.009	**0**.**742**
LGLE	2.980 × 10^−3^	0.0025–0.0032	3.600 × 10^−3^	0.0029–0.0041	0.013	**0**.**729**
RLNU	5.040× 10^5^	4.83 × 10^5^–5.91 × 10^5^	4.480 × 10^5^	4.11 × 10^5^–5.09 × 10^5^	0.013	**0**.**729**
Kurtosis	2.720 × 10	2.565–2.862	2.963 × 10	2.759–3.154	0.018	**0**.**718**
LDLGLE	3.610 × 10	2.458–4.332	4.774 × 10	3.327–6.721	0.020	**0**.**716**
LRLGLE	3.810 × 10^−3^	0.0031–0.0042	4.700 × 10^−3^	0.004–0.014	0.024	**0**.**709**
MP	8.000 × 10^−5^	6.46 × 10^−5^–9.99 × 10^−5^	1.100 × 10^−4^	8.55 × 10^−5^−1.57 × 10^−4^	0.027	**0**.**704**
LGLE	7.700 × 10^−4^	7.04 × 10^−4^–9.19 × 10^−4^	9.800 × 10^−4^	7.86 × 10^−4^−1.28 × 10^−3^	0.028	**0**.**702**
LALGLE	7.000 × 10^−5^	6.57 × 10^−5^–9.12 × 10^−5^	1.000 × 10^−4^	7.37 × 10^−5^–0.245	0.032	**0**.**698**
GLNU	9.960 × 10^3^	9.02 × 10^3^–1.15 × 10^4^	8.640 × 10^3^	7.88 × 10^3^−1.01 × 10^4^	0.032	**0**.**698**
Energy	3.770 × 10^10^	3.26 × 10^10^–4.92 × 10^10^	3.380 × 10^10^	2.67 × 10^10^–4.02 × 10^10^	0.041	**0**.**689**
Total Energy	4.710 × 10^9^	4.08 × 10^9^–6.15 × 10^9^	4.220 × 10^9^	3.34 × 10^9^–5.03 × 10^9^	0.041	**0**.**689**
Coarseness	9.000 × 10^−6^	8.05 × 10^−6^–1.01 × 10^−5^	9.929 × 10^−6^	9.24 × 10^−6^−1.13 × 10^−5^	0.041	**0**.**689**
SRLGLE	1.900 × 10^−4^	0.00018–0.00024	2.600 × 10^−4^	0.00020–0.00038	0.043	**0**.**687**
Skewness	2.060 × 10^−1^	0.107–0.382	3.480 × 10^−1^	0.210–0.467	0.102	0.651
ZV	2.5 × 10	1.527–3.835	3.006 × 10	2.276–6.142	0.219	0.613
CS	2.201 × 10^2^	90.25–968.56	5.375 × 10^2^	281.57–855.78	0.219	0.613
MCC	5.500 × 10^−1^	0.505–0.681	6.300 × 10^−1^	0.557–0.734	0.301	0.596
CP	2.870 × 10^8^	1.32 × 10^8^–3.83 × 10^8^	1.930 × 10^8^	1.60 × 10^8^–2.38 × 10^8^	0.485	0.564
SDLGLE	2.200 × 10^−4^	1.83 × 10^−4^–3.09 × 10^−4^	2.100 × 10^−4^	1.36 × 10^−4^–3.03 × 10^−4^	0.485	0.564
Busyness	2.402 × 10^2^	203.26–310.05	2.400 × 10^2^	200.54–289.02	0.647	0.542
FRAX overall + BMD	2.000 × 10^1^	14.5–27	1.200 × 10^1^	9.125–19.75	0.008	**0**.**746**
FRAX-Hip + BMD	4.300 × 10	2.9–8.3	2.500 × 10	1.825–4.375	0.043	**0**.**687**
FRAX overall	2.100 × 10^1^	17–27.5	1.100 × 10^1^	8.775–17.75	0.002	**0**.**789**
FRAX-Hip	5.100 × 10	3.5–9.25	2.400 × 10	1.4–3.95	0.008	**0**.**747**
T-score (Femoral Neck)	−2.600 × 10	−8.000 × 10^−1^	−2.400 × 10	(−2.8)-(−2.2)	0.866	0.516
T-score (Hip)	−2.400 × 10	−5.000 × 10^−1^	−2.300 × 10	(−2.5)-(−1.9)	0.397	0.585

IRQ, interquartile range.

Significant differences are highlighted in bold (*p* < 0.05).

Radiomic features could discriminate between Fx and nFx with AUROC values ranging from 0.687–0.751 (*p*-value < 0.05). The non-uniformity features DNU, SZNU and RLNU showed high discriminatory ability (AUROC > 0.7; *p*-value < 0.014); the first order features such as Kurtosis showed significant discriminatory ability (AUROC = 0.718; *p*-value = 0.0183). Furthermore, GLDM and GLRLM features including LGLE, LDLGE and LRLGLE measure the emphasis on low gray level pixels and large accumulation of similar pixel intensity within neighborhood in the trabecular ROI and showed significant discriminatory ability (AUROC > 0.7; *p*-value < 0.024) between Fx and nFx group.

FRAX scores could discriminate between Fx and nFx group with AUROC values ranging from 0.687–0.745. *T*-scores could not discriminate between Fx and nFx groups which was expected since there was no significant difference in *T*-scores between the groups. DNU and SZNU had AUROC values comparable to those of FRAX scores.

### Relationship between radiomic features and clinical parameters.

Correlation between the radiomic features and clinical parameters are presented in Table 4. Non-Uniformity features such as DNU, SZNU and RLNU demonstrated a significantly weak to moderate positive correlation with FRAX (0.299–0.444). DNU, SZNU, RLNU and GLNU demonstrated a moderate positive correlation with FRAX + BMD measure (*ρ *= 0.461, 0.484, 0.531 and 0.527 respectively). First order features such as kurtosis showed no correlation with age, FRAX and *T*-scores. Moreover, GLDM and GLRLM features such as LGLE, LDLGLE and LRLGLE showed no significant correlation with any clinical metrics or FRAX scores.

**Table 4:** Spearman correlation between radiomic features and clinical parameters.

Features	Tscore (Hip)	Age	FRAX + BMD	FRAX	FRAX-Hip + BMD	FRAX-Hip	Tscore (Femoral neck)
DNU	−0.330	0.012	**0**.**461**	0.299	0.196	0.063	−0.105
SZNU	**−0**.**366**	0.061	**0**.**484**	0.315	0.238	0.082	−0.158
LGLE	0.067	0.256	−0.082	−0.193	0.097	−0.121	−0.004
RLNU	−0.304	0.075	**0**.**531**	**0**.**444**	0.275	0.230	−0.113
Kurtosis	−0.050	−0.094	−0.190	−0.223	−0.240	−0.217	0.085
LDLGLE	0.042	0.253	0.054	−0.002	0.129	0.064	−0.130
LRLGLE	0.055	0.297	0.041	−0.119	0.167	−0.012	0.091
MP	0.069	0.260	0.096	0.012	0.169	0.194	0.149
LGLRE	0.011	0.289	0.023	−0.121	0.144	−0.048	0.081
LALGLE	0.113	0.190	0.103	−0.152	0.226	−0.089	−0.070
GLNU	−0.277	0.088	**0**.**527**	**0**.**362**	**0**.**356**	0.189	−0.315
Energy	−0.072	**0**.**355**	0.307	**0**.**361**	0.249	**0**.**356**	−0.012
TE	−0.072	**0**.**355**	0.307	**0**.**361**	0.249	**0**.**356**	−0.012
Coarseness	0.077	−0.084	−0.285	−0.286	0.054	−0.118	0.124
SRLGLE	−0.009	0.314	0.046	−0.120	0.136	−0.044	0.092
Skewness	−0.094	−0.157	**−0**.**308**	**−0**.**339**	−0.201	−0.279	−0.082
ZV	−0.034	0.315	0.131	0.020	0.216	**0**.**382**	−0.073
CS	−0.075	−0.090	**−0**.**322**	**−0**.**350**	−0.220	−0.279	0.082
MCC	−0.046	0.289	0.045	0.076	0.128	**0**.**330**	0.220
CP	−0.048	0.042	−0.240	−0.126	−0.238	−0.065	**0**.**342**
SDLGLE	−0.169	−0.251	−0.300	−0.263	−0.150	**−0**.**403**	−0.158
Busyness	−0.138	−0.079	0.243	0.043	0.151	−0.062	**−0**.**322**

Significant correlation values are highlighted in bold (*p*-value < 0.05).

### Correlation between features

**Figure 2:**
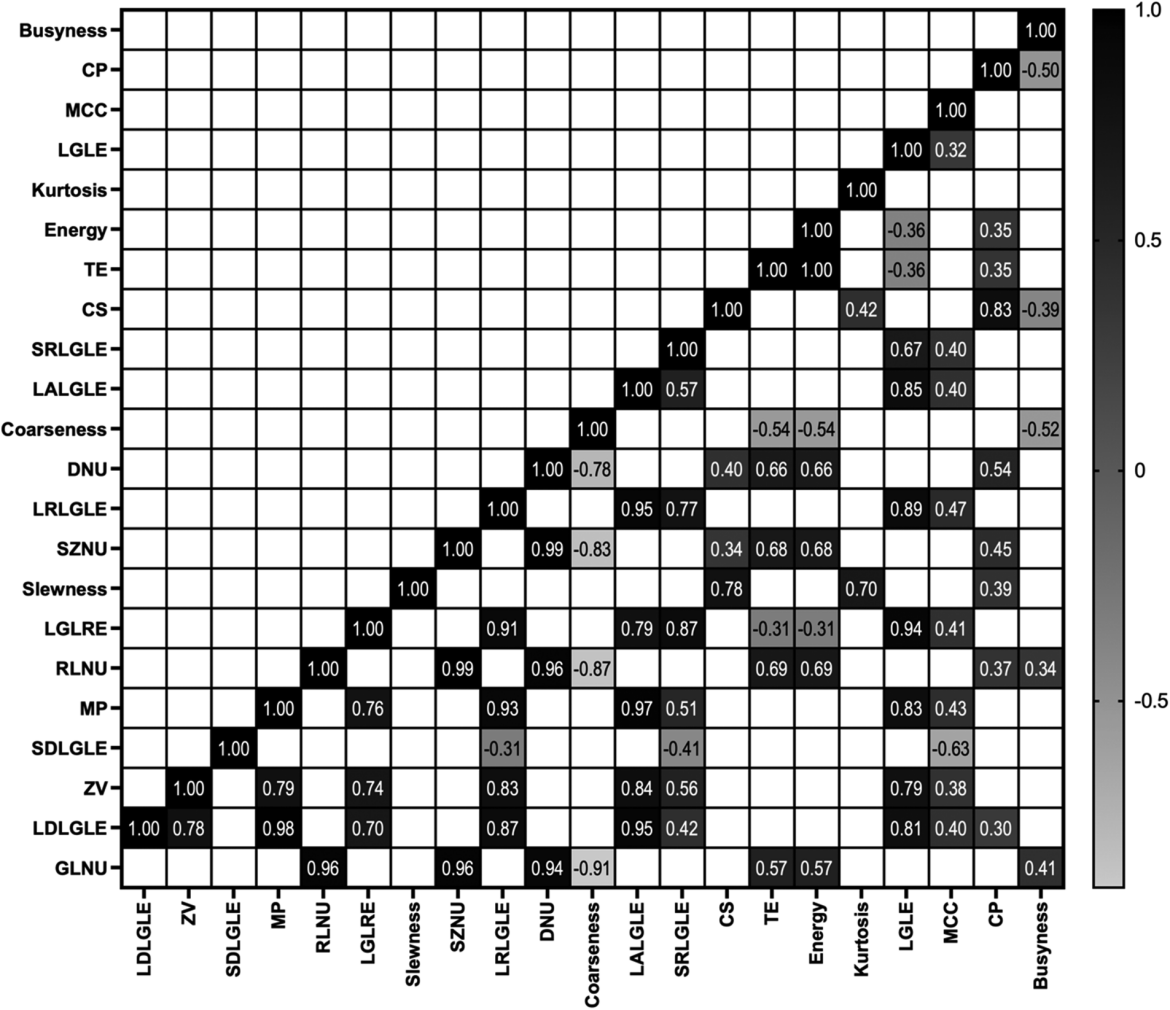
Significant correlation found between features using Pearson's r coefficient (*p*-value < 0.01).

Figure 2 shows the Pearson-correlation between radiomic features. Among the features that showed high ability to predict fragility fracture were SZNU, DNU, and RLNU—or coarseness features—and these were significantly and highly correlated with each other. LGLE, LDLGLE, MP and LRLGLE, which also showed significant ability to predict fragility fracture, were also significantly and highly correlated with each other.

## Discussion/conclusion

In conclusion, MRI-based radiomics can discriminate between OP women with and without fragility fractures. According to our analysis, we identified that DNU, LGLE and Kurtosis are three features of interest since they have the highest AUROC, very low correlation with other radiomic features, and weak correlation with BMD and FRAX scores suggesting that they could be used as novel, imaging biomarkers for bone health that provide complementary information to each other and to DXA and FRAX.

In the T1-contrast MRI acquisitions of the trabecular region of the proximal femur used in this study, subjects with fragility fracture showed lower kurtosis and LGLE values and higher DNU values compared to subjects without fracture. DNU and LGLE are local second-order features. DNU measures nonuniformity in the interdependence of pixels within the trabecular region of interest in the proximal femur. LGLE measures the empasis of low intensity pixels within the trabecular region of interest. Kurtosis is a global first-order feature. High kurtosis values imply that in the region of interest there is a copmaratively large number of pixel values towards the extremes, while a low kurtosis value implies high peakedness of the distribution. MRI of microarchitecture indirectly image of the trabeculae since it relies on the contrast between non fully relaxed bone marrow fat tissue and trabeculae fully relaxed signals. GLDM features that we found of interest are defined on low intensity voxels, notably their dependance (LGLE) and their uniformity (DNU). They may correspond to a measure of the trabecular bone network since in our image fat appear hyperintense and bone hypointense. One of the technical considerations in our study was the MRI image resolution, specifically the slice direction resolution being much lower than the in-plane resolution. Differences in resolution can potentially influence the granularity of the features extracted and might play a role in the robustness of the radiomic analysis. In the context of bone health and fracture risk assessment, where subtle variations in the trabecular structure can be critical, the resolution of the MRI images can be a determining factor. While our study utilized high-resolution MR images of the proximal femur to ensure detail preservation, it is imperative for future research to investigate the direct impact of varying resolutions, especially slice direction resolution, on radiomic feature extraction and subsequent analyses. Such investigations would provide clearer insights into the optimal imaging parameters for robust radiomic analyses in osteoporosis assessments.

In recent years, several studies have investigated the use of radiomics in oncology to analyse tumors. There are few studies which have investigated the use of radiomics for bone health assessment. In the lumbar spine a multi-contrast approach was evaluated using both T1 and T2 weighted MRI (23) to detect osteoporosis compared to osteopenic and controls subjects. They found similar AUROC values of 0.73 using T1-weighted images, of 0.734 for T2 weighted image, and of 0.769 when using T1- and T2-weighted images. Another study used opportunistic abdominal CT to retrospectively compute 41 radiomic features in the proximal femur of 500 patients (24) to predict OP status and found an AUROC of 0.96 to predict OP. More recent studies combine radiomic features computed using CT and MRI scans of the lumbar spine (25). They notably used chemical shift encoded MRI methods to separate bone marrow from MR images. They showed that the use of additional radiomic features can provide a better differentiation between OP patients with and without vertebral fracture (47% of the variance in osteoporotic vertebral fracture was explained by the model when it was based on BMD and bone marow measurement only compared to 81% of the variance in fracture when adding textural features to the model).

To the best of our knowledge, our study is the first to analyze the relationship between high-resolution MR-based radiomic features of the proximal femur and osteoporotic fracture status and the relationship between MR-based radiomic features and standard-of care measures of fracture risk such as FRAX and BMD.

In this study, we decided to perform a univariate analysis. Most studies use multiple combinations of feature selection and machine learning models over unmatched datasets to arrive at a model that shows high predictive ability. However, while advanced ML models have feature selection capabilities, our study's objective was to provide foundational insights into the underlying radiomics features critical for osteoporotic fracture risk. The distinct features we identified could guide and refine the feature selection process in subsequent ML models, ensuring they're both statistically robust and grounded in domain-specific knowledge. The limitation of such methods is that high predictivity might be influenced by confounding factors, such as age or BMI. Matching the test and control data has the effect of limiting the size of the dataset. In earlier stage of this study we used the above defined method but found that during feature selection methods one or two features were selected and individual features showed similar predictive ability as models with several features. Features showing significant predictive ability could be deciphered by curating the dataset by using high quality MRI images with matched control and test groups. Measuring and analyzing the predictive accuracy of individual features could help to use the dataset more effectively rather than building a multiparametric machine learning model.

The correlation between radiomics parameters and traditional indicators such as FRAX/BMD is weak to moderate. This suggests that radiomics parameters provide information distinct from that captured by FRAX/BMD. If there was a strong correlation, it would mean the information from both sources overlaps, reducing the need to assess radiomics parameters. However, the observed correlation indicates that radiomics parameters capture different aspects of bone health.

Given this distinction, while FRAX and BMD remain primary tools for osteoporosis screening, radiomics analyses might be used for further risk stratification. This approach could be particularly beneficial when primary screening results are inconclusive.This study is not without limitations. The first limitation is the relativley small size of the dataset. However, as an initial pilot study, we believe that this is sufficient in size and provides the foundation and evidence for a larger study with more fracture cases and controls. Second, with larger dataset, we could build machine learning models combining multiple features to predict osteoporotic fracture risk. This would be possible now that we know which types of features are most important after doing the univariate analyses in this study. Third, we do not have clinical imaging or microarchitectural information on these subjects, and in the future it would be important to determine the correlation between radiomic information and microarchitectural parameters or information that could be derived from clinical scans. Finally, this study is limited in that we used a T1-weighted FLASH acquisition for the MRI data. Moreover, considering the potential of MR Fingerprinting (MRF) as a quantitative method for assessing fracture risk, future research might benefit from exploring its utility alongside traditional imaging techniques. In the future, it would be important to investigate the effect of different types of image acquisitions or even investigate MRF in more depth as a promising method to assess fracture risk.

In conclusion, we have shown that MR-based radiomics of the proximal femur, in particular the features of DNU, LGLE, and Kurtosis can discriminate osteoporotic fracture cases from controls and provides different information about fracture risk compared to DXA and FRAX. Larger, longitudinal studies are need to help determine whether these radiomics parameters could have value to predict future fracture.

## Data Availability

The raw data supporting the conclusions of this article will be made available by the authors, without undue reservation.
